# Supplementary material for: Factors that influence older patients’ participation in clinical communication within developed country hospitals and GP clinics: A systematic review of current literature
Source: PLoS One. 2022 Jun 27;17(6):e0269840. doi: 10.1371/journal.pone.0269840 (PMC9236261; doi:10.1371/journal.pone.0269840)
Supplement: S1 Protocol — https://www.crd.york.ac.uk/PROSPEROFILES/164716_PROTOCOL_20220124.pdf. (PDF) [file pone.0269840.s002.pdf]

Systematic review

1. * Review title.

Give the title of the review in English

Factors that influence older patients' participation in clinical communication within developed country hospitals and GP Clinics: A systematic review of current literature.

2. Original language title.

For reviews in languages other than English, give the title in the original language. This will be displayed with the English language title.

English

3. * Anticipated or actual start date.

Give the date the systematic review started or is expected to start.

23/09/2019

4. * Anticipated completion date.

Give the date by which the review is expected to be completed.

31/12/2021

5. * Stage of review at time of this submission.

Tick the boxes to show which review tasks have been started and which have been completed. Update this field each time any amendments are made to a published record.

Reviews that have started data extraction (at the time of initial submission) are not eligible for inclusion in PROSPERO. If there is later evidence that incorrect status and/or completion date has been supplied, the published PROSPERO record will be marked as retracted.

This field uses answers to initial screening questions. It cannot be edited until after registration.

The review has not yet started: No

Review stage	Started	Completed
Preliminary searches	Yes	Yes
Piloting of the study selection process	Yes	Yes
Formal screening of search results against eligibility criteria	Yes	Yes
Data extraction	Yes	Yes
Risk of bias (quality) assessment	Yes	Yes
Data analysis	Yes	Yes

Provide any other relevant information about the stage of the review here.

6. * Named contact.

The named contact is the guarantor for the accuracy of the information in the register record. This may be any member of the review team.

Harry Gaffney

Email salutation (e.g. "Dr Smith" or "Joanne") for correspondence:

Mr Harry Gaffney

7. * Named contact email.

Give the electronic email address of the named contact.

harry.gaffney@flinders.edu.au

harryjamesgaffney@gmail.com

8. Named contact address

Give the full institutional/organisational postal address for the named contact.

College of Medicine and Public Health, Flinders University Flinders University, Adelaide, South Australia

9. Named contact phone number.

Give the telephone number for the named contact, including international dialling code.

0410818754

10. * Organisational affiliation of the review.

Full title of the organisational affiliations for this review and website address if available. This field may be completed as 'None' if the review is not affiliated to any organisation.

Flinders University

Organisation web address:

11. * Review team members and their organisational affiliations.

Give the personal details and the organisational affiliations of each member of the review team. Affiliation refers to groups or organisations to which review team members belong. **NOTE: email and country now MUST be entered for each person, unless you are amending a published record.**

Mr Harry Gaffney. Flinders University
Mohammad Hamiduzzaman. Flinders University

12. * Funding sources/sponsors.

Details of the individuals, organizations, groups, companies or other legal entities who have funded or sponsored the review.

Harry Gaffney, Mohammad Hamiduzzaman

Grant number(s)

State the funder, grant or award number and the date of award

13. * Conflicts of interest.

List actual or perceived conflicts of interest (financial or academic).

None

14. Collaborators.

Give the name and affiliation of any individuals or organisations who are working on the review but who are not listed as review team members. **NOTE: email and country must be completed for each person, unless you are amending a published record.**

15. * Review question.

State the review question(s) clearly and precisely. It may be appropriate to break very broad questions down into a series of related more specific questions. Questions may be framed or refined using PI(E)COS or similar where relevant.

What are the influencers to clinical communication participation from the older patient's perspective?

16. * Searches.

State the sources that will be searched (e.g. Medline). Give the search dates, and any restrictions (e.g. language or publication date). Do NOT enter the full search strategy (it may be provided as a link or attachment below.)

Academic journal databases: CINAHL, Cochrane, EmCare, MEDLINE, PsycINFO, Scopus, Web of

Science, ProQuest

Other databases: Google Scholar

Date searched (all): 04/01/2020

Restrictions: English articles published from 2010 onwards only

17. URL to search strategy.

Upload a file with your search strategy, or an example of a search strategy for a specific database, (including the keywords) in pdf or word format. In doing so you are consenting to the file being made publicly accessible. Or provide a URL or link to the strategy. Do NOT provide links to your search **results**.

https://www.crd.york.ac.uk/PROSPEROFILES/164716_STRATEGY_20200108.pdf

Alternatively, upload your search strategy to CRD in pdf format. Please note that by doing so you are consenting to the file being made publicly accessible.

Do not make this file publicly available until the review is complete

18. * Condition or domain being studied.

Give a short description of the disease, condition or healthcare domain being studied in your systematic review.

This review will undertake a thematic synthesis of the challenges experienced in clinical communications from the perspective of Elderly patients. In doing so, areas of opportunity to improve doctor-patient communications may be identified and health outcomes for the elderly may be improved.

19. * Participants/population.

Specify the participants or populations being studied in the review. The preferred format includes details of both inclusion and exclusion criteria.

The elderly population (65 and over) and health professionals including General Practitioners, nurses, and all affiliates directly involved in patient clinical communications in developed countries.

20. * Intervention(s), exposure(s).

Give full and clear descriptions or definitions of the interventions or the exposures to be reviewed. The preferred format includes details of both inclusion and exclusion criteria.

The focus of this review is to provide a comprehensive account of the experiences in clinical communications from the perspective of Elderly patients. This includes factors such as poor clinician verbal and non-verbal communication (e.g. lack of person-centred care), elderly patients not understanding how to prepare for appointments and cultural barriers.

21. * Comparator(s)/control.

Where relevant, give details of the alternatives against which the intervention/exposure will be compared (e.g. another intervention or a non-exposed control group). The preferred format includes details of both inclusion and exclusion criteria.

Not applicable

22. * Types of study to be included.

Give details of the study designs (e.g. RCT) that are eligible for inclusion in the review. The preferred format includes both inclusion and exclusion criteria. If there are no restrictions on the types of study, this should be stated.

Qualitative, qualitative and mixed-method studies.

23. Context.

Give summary details of the setting or other relevant characteristics, which help define the inclusion or exclusion criteria.

24. * Main outcome(s).

Give the pre-specified main (most important) outcomes of the review, including details of how the outcome is defined and measured and when these measurement are made, if these are part of the review inclusion criteria.

To identify the clinical communication experiences of older people and (1) highlight any barriers that could be addressed or (2) facilitators that could be reinforced.

Measures of effect

Please specify the effect measure(s) for you main outcome(s) e.g. relative risks, odds ratios, risk difference, and/or 'number needed to treat.

Not applicable

25. * Additional outcome(s).

List the pre-specified additional outcomes of the review, with a similar level of detail to that required for main outcomes. Where there are no additional outcomes please state 'None' or 'Not applicable' as appropriate to the review

Identify further research that could be undertaken to improve elderly patients experiences while navigating the healthcare system.

To uncover how the Elderly population communication experiences with health professionals vary depending on the health professional's geographic location.

Measures of effect

Please specify the effect measure(s) for you additional outcome(s) e.g. relative risks, odds ratios, risk difference, and/or 'number needed to treat.

Not applicable

26. * Data extraction (selection and coding).

Describe how studies will be selected for inclusion. State what data will be extracted or obtained. State how this will be done and recorded.

One reviewer (Harry Gaffney) will independently review each title/abstract identified during the literature search to determine if the study is a rejected (i.e. the full text will not be reviewed because it does not fit review inclusion criteria) or a "maybe" (i.e. further reviewed to determine if the study fits the inclusion criteria). In these cases, the full text will be read, and a decision will be made to exclude or potentially include the article.

Full-text articles will then be obtained for the potentially included articles. An independent assessment will then be performed by the original reviewer (Harry Gaffney) and a secondary reviewer, who will determine if they meet the criteria for inclusion in the final systematic review. Upon completion, a meeting will be held to compare, discuss, and justify the independent assessments. The meetings aim will be to ultimately determine the articles final selection to be included in the systematic literature review.

27. * Risk of bias (quality) assessment.

State which characteristics of the studies will be assessed and/or any formal risk of bias/quality assessment

tools that will be used.

A data table will be established that includes relevant information for data extraction including study population demographics and size, study methodology, recruitment, outcomes of interest, findings and any identified limitations and potential bias sources.

28. * Strategy for data synthesis.

Describe the methods you plan to use to synthesise data. This **must not be generic text** but should be **specific to your review** and describe how the proposed approach will be applied to your data. If meta-analysis is planned, describe the models to be used, methods to explore statistical heterogeneity, and software package to be used.

The data from all included studies will be analysed utilising a thematic synthesis methodology. Any outcomes of interest, study findings and their similarities and differences will be highlighted. Similarities in how the elderly populations experiences in clinical communications will also be highlighted. Any gaps in current knowledge will also be identified.

29. * Analysis of subgroups or subsets.

State any planned investigation of ‘subgroups’. Be clear and specific about which type of study or participant will be included in each group or covariate investigated. State the planned analytic approach.

None planned.

30. * Type and method of review.

Select the type of review, review method and health area from the lists below.

Type of review

Cost effectiveness
No

Diagnostic
No

Epidemiologic
No

Individual patient data (IPD) meta-analysis
No

Intervention
No

Living systematic review
No

Meta-analysis
No

Methodology
No

Narrative synthesis
No

Network meta-analysis

No

Pre-clinical
No

Prevention
No

Prognostic
No

Prospective meta-analysis (PMA)
No

Review of reviews
No

Service delivery
No

Synthesis of qualitative studies
No

Systematic review
Yes

Other
No

Health area of the review

Alcohol/substance misuse/abuse
No

Blood and immune system
No

Cancer
No

Cardiovascular
No

Care of the elderly
No

Child health
No

Complementary therapies
No

COVID-19
No

Crime and justice
No

Dental
No

Digestive system
No

Ear, nose and throat
No

Education
No

Endocrine and metabolic disorders
No

Eye disorders
No

General interest
No

Genetics
No

Health inequalities/health equity
No

Infections and infestations
No

International development
No

Mental health and behavioural conditions
No

Musculoskeletal
No

Neurological
No

Nursing
No

Obstetrics and gynaecology
No

Oral health
No

Palliative care
No

Perioperative care
No

Physiotherapy
No

Pregnancy and childbirth
No

Public health (including social determinants of health)
No

Rehabilitation
No

Respiratory disorders

No

Service delivery
Yes

Skin disorders
No

Social care
No

Surgery
No

Tropical Medicine
No

Urological
No

Wounds, injuries and accidents
No

Violence and abuse
No

31. Language.

Select each language individually to add it to the list below, use the bin icon to remove any added in error.
English

There is not an English language summary

32. * Country.

Select the country in which the review is being carried out. For multi-national collaborations select all the countries involved.

Australia

33. Other registration details.

Name any other organisation where the systematic review title or protocol is registered (e.g. Campbell, or The Joanna Briggs Institute) together with any unique identification number assigned by them. If extracted data will be stored and made available through a repository such as the Systematic Review Data Repository (SRDR), details and a link should be included here. If none, leave blank.

34. Reference and/or URL for published protocol.

If the protocol for this review is published provide details (authors, title and journal details, preferably in Vancouver format)

Add web link to the published protocol.

Or, upload your published protocol here in pdf format. Note that the upload will be publicly accessible.

No I do not make this file publicly available until the review is complete

Please note that the information required in the PROSPERO registration form must be completed in full even if access to a protocol is given.

35. Dissemination plans.

Do you intend to publish the review on completion?

Yes

Give brief details of plans for communicating review findings.?

36. Keywords.

Give words or phrases that best describe the review. Separate keywords with a semicolon or new line. Keywords help PROSPERO users find your review (keywords do not appear in the public record but are included in searches). Be as specific and precise as possible. Avoid acronyms and abbreviations unless these are in wide use.

Clinical communication, older patients, Hospitals, GP clinics, disposing factors.

37. Details of any existing review of the same topic by the same authors.

If you are registering an update of an existing review give details of the earlier versions and include a full bibliographic reference, if available.

38. * Current review status.

Update review status when the review is completed and when it is published.New registrations must be ongoing so this field is not editable for initial submission.
Please provide anticipated publication date

Review_Ongoing

39. Any additional information.

Provide any other information relevant to the registration of this review.

40. Details of final report/publication(s) or preprints if available.

Leave empty until publication details are available OR you have a link to a preprint (NOTE: this field is not editable for initial submission). List authors, title and journal details preferably in Vancouver format.

Give the link to the published review or preprint.
