# Supplementary material for: Factors that influence older patients’ participation in clinical communication within developed country hospitals and GP clinics: A systematic review of current literature
Source: PLoS One. 2022 Jun 27;17(6):e0269840. doi: 10.1371/journal.pone.0269840 (PMC9236261; doi:10.1371/journal.pone.0269840)
Supplement: S1 Text — (DOCX) [file pone.0269840.s003.docx]

The following is an example of a search string applied to the CINAHL database: ("old* people" OR elderly OR geriatric* OR senior).kf. AND (patient* N4 (interact* OR satisf* OR experienc* OR attitude* OR view* OR belief* OR believe* OR prefer* OR perception OR perspective OR need*) N2 ("clinical practice" OR healthcare* OR health* OR physician* OR "primary care" OR GPs OR "general pract*" OR doctor* OR "health personnel*" OR "health profession*").kf. AND (communicat* OR negotiat* OR "patient-centred care" OR "patient-centered care" OR "person-centred care" OR "person-centered care" OR "client-centred care" OR "client-centered care" OR "patient centred care" OR "patient centered care" OR "person centred care" OR "person centered care" OR "client centred care" OR "client centered care" OR "social skill*" OR "patient decision making" OR "information exchang*" OR trust OR "patient navigat*").kf. AND (Australia* OR Queensland* OR New south wales OR victoria* OR tasmania* OR northern territory* OR New Zealand* OR canad* OR alberta* OR british columbia* OR manitoba* OR new brunswick* OR newfoundland* OR labrador* OR northwest territories OR nova scotia* OR nunavut* OR ontario* OR prince edward island* OR quebec* OR saskatchewan* OR yukon territory* OR Britain* OR British OR United Kingdom* OR England* OR English OR Scotland* OR Scottish* OR Wales OR Welsh OR Channel Islands OR Northern Ireland* OR Irish OR united states OR North america* OR USA OR Scandinavia* OR nordic OR denmark* OR danish OR greenland* OR finland* OR finnish OR iceland* OR norway* OR norwegian* OR sweden* OR swedish OR Belgium OR France OR Monaco OR Netherlands OR Austria OR Germany OR Switzerland OR Greece OR Italy).mp.
