# Supplementary material for: Factors that influence older patients’ participation in clinical communication within developed country hospitals and GP clinics: A systematic review of current literature
Source: PLoS One. 2022 Jun 27;17(6):e0269840. doi: 10.1371/journal.pone.0269840 (PMC9236261; doi:10.1371/journal.pone.0269840)
Supplement: Attachment — Submitted filename: 1. Response to Reviewers 190322.docx [file pone.0269840.s005.docx]

**Responses to Journal requirements**

**Reviewers' comments:**

The authors have responded to the vast majority of the observations I have raised in a manner that enriches the research, but I believe that there is still a need for a slight additional revision of the grammatical and language used and the punctuation between the phrases and in the sentences used in the manuscript.

Also, you can use these references:

1. Bahadori, M., Hasanpoor, E., Yaghoubi, M. and HaghGoshyie, E., 2020. Determinants of a high-quality consultation in medical communications: a systematic review of qualitative and quantitative evidence. *International Journal of Human Rights in Healthcare*.
2. Hajebrahimi S, Janati A, Arab-Zozani M, Sokhanvar M, Haghgoshayie E, Siraneh Y, Bahadori M, Hasanpoor E. Medical visit time and predictors in health facilities: a mega systematic review and meta-analysis. International Journal of Human Rights in Healthcare. 2019 Nov 28.
3. Bahadori M, Yaghoubi M, Haghgoshyie E, Ghasemi M, Hasanpoor E. Patients’ and physicians’ perspectives and experiences on the quality of medical consultations: a qualitative study. JBI Evidence Implementation. 2020 Jun 1;18(2):247-55.
4. Hasanpoor E, Bahadori M, Yaghoubi M, Haghgoshayie E, Mahboub-Ahari A. Evidence-based management as a basis for evidence-based medical consultation. BMJ evidence-based medicine. 2020 Jun 1;25(3):83-4.

**Response:**

Additional revision has been conducted to address the readability, grammar and language used. This also includes the punctuation between the phrases and in the sentences used in the manuscript.

This revision has been done so to achieve a closer alignment with the source references supplied by the reviewer.
